# Reaching multidisciplinary consensus on classification of anaphylaxis for the eleventh revision of the World Health Organization's (WHO) International Classification of Diseases (ICD-11)

**DOI:** 10.1186/s13023-017-0607-3

**Published:** 2017-03-16

**Authors:** Luciana Kase Tanno, Robert J. G. Chalmers, Moises A. Calderon, Ségolène Aymé, Pascal Demoly, on behalf the Joint Allergy Academies

**Affiliations:** 1 Hospital Sírio Libanês, São Paulo, Brazil; 2 Division of Allergy, Department of Pulmonology, Hôpital Arnaud de Villeneuve, University Hospital of Montpellier, 371, av. du Doyen Gaston Giraud, 34295 Montpellier cedex 5, France; 3 Sorbonne Universités, UPMC Paris 06, UMR-S 1136, IPLESP, Equipe EPAR, 75013 Paris, France; 4 Dermatology Topic Advisory Group, ICD-11 Revision Steering Group and Honorary Consultant Dermatologist, University of Manchester, Manchester, UK; 5 Section of Allergy and Clinical Immunology, Imperial College London, National Heart and Lung Institute, Royal Brompton Hospital, London, UK; 6 INSERM, US14, Paris, France

**Keywords:** Anaphylaxis, Classification, International Classification of Diseases, World Health Organization

## Abstract

**Background:**

Although currently misclassified in the International Classification of Diseases (ICD) and still not officially listed as a rare disease, anaphylaxis is a well-known clinical emergency. Anaphylaxis is now one of the principal headings in the “Allergic and hypersensitivity conditions” section recently compiled for the forthcoming 11^th^ Revision of ICD (ICD-11). We here report the building process used for the pioneering “Anaphylaxis” subsection of ICD-11 in which we aimed for transparency as recommended in the ICD-11 revision guidelines.

**Results:**

During an online intensive scientific and technical discussions with ICD-11 Topic Advisory Groups and Expert Working Groups, we drafted a total of 35 proposals for the classification of anaphylaxis. From all the 35 proposals, 77% were implemented, 20% remain to be implemented, and the others being partially implemented (1.5%) or rejected (1.5%).

**Conclusion:**

For the first time, anaphylaxis is now properly classified and has attained greater visibility within ICD. In addition to all the benefits expected from the actions we have undertaken in updating the terminology, definitions and classification of allergic and hypersensitivity conditions for ICD-11, we strongly believe that anaphylaxis should be a public health priority and that it should therefore be formally added into the list of rare diseases in order to support awareness and quality clinical management of patients.

## Background

### Anaphylaxis as a rare disease: definitions, epidemiology and unmet needs

**Fig. 1:**
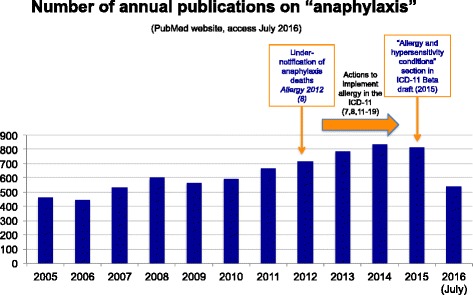
Annual number of publications retrieved from Pubmed Central® using the search term “anaphylaxis”, (accessed July 2016)

Since the term “anaphylaxis” was first coined by Charles Richet and Paul Portier [1], it has rapidly spread all over the world and its clinical importance as an emergency condition is now well accepted. Anaphylaxis is nowadays recognized as a severe, life-threatening systemic hypersensitivity reaction characterized by rapid onset and the potential to endanger life through airway, breathing, or circulatory problems. It is usually, although not always, associated with skin and mucosal changes [2–4]. This multi-faceted condition can manifest at any age and any health professional may be faced by it. In recent years there has been an increasing number of publications aimed at heightening awareness of this issue (Fig. 1).

Regional epidemiological data cite anaphylaxis incidence rates ranging from 1.5 to 7.9 per 100 000 person-years in European countries [4] and estimated in at 5.1% (95% CI, 3.4 to 6.8%) in the United States [5]. Based on these statistics, anaphylaxis would fit well the definition of a rare disease, although it is not currently listed in rare diseases registries [6]. The epidemiological criteria for designating a condition a “rare disease” vary depending on the reference in consideration but, conceptually, rare diseases can be defined as life-threatening or chronic debilitating disorders which are of low prevalence and typically require combined efforts to address them. The global epidemiological morbidity [7] and mortality [8] data for anaphylaxis remain unclear due to the lack of standardized tools for capturing harmonized and accurate data, particularly in the International Classification of Diseases, Injuries and Cause of Death (ICD). This fact has a direct impact on the awareness it receives for healthcare planning and resource allocation, quality patient management and public health policy.

### Anaphylaxis in the International Classification of Diseases

The ICD is a global standard diagnostic classification for mortality and morbidity statistics maintained by the World Health organization (WHO). Currently the majority of countries use the tenth revision of ICD (ICD-10) or adaptations thereof [9]. ICD-10 has inherited a structure from previous versions of ICD in which topographic distribution frequently takes precedence over underlying mechanisms, triggers or any of the concepts currently used for allergic and hypersensitivity conditions. As a result, only two terms in ICD-10 for anaphylaxis are hidden within section T78 of *Other and unspecified effects of external causes* under the unsatisfactory title *Adverse effects, not elsewhere classified* [10]. The inadequacy of this classification is a major reason for the under-notification of anaphylaxis in vital statistics [8].

**Fig. 2:**
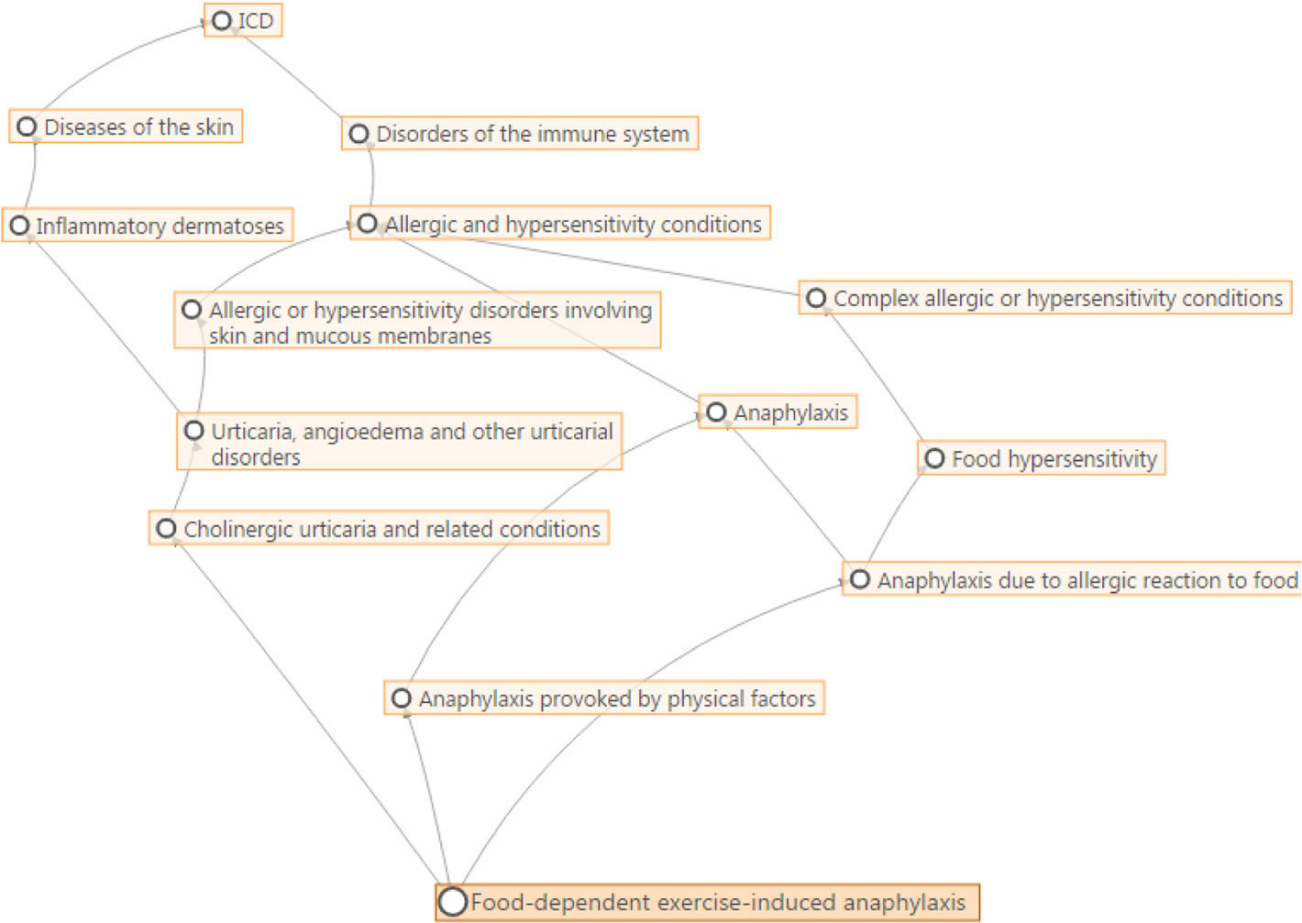
Example of ICD-11 online polyhierarchical Foundation framework taken the search for the term “Food-dependent exercise-induced anaphylaxis”

In development since 2007, ICD-11 is intended not only to rectify deficiencies in ICD-10 and to incorporate changes demanded by scientific advances, but also to take advantage of the revolution in electronic data handling since the publication of ICD-10 a quarter of a century ago. ICD-11 may be regarded as a suite of classifications which is based on a detailed and comprehensive polyhierarchical web-like Foundation (Fig. 2) in which any single disease entity may be represented in more than one location.

From this Foundation may be extracted any number of traditional tabular lists, which differ from the Foundation in that a single entity may appear in only one location (as in ICD-10 and, as in the latter’s proposed replacement, the ICD-11 for Mortality and Morbidity Statistics (ICD-11 MMS)). This will also permit the construction of a range of specialist classifications in which the detail contained in the Foundation is retained but which can link to ICD-11 MMS.

**Fig. 3:**
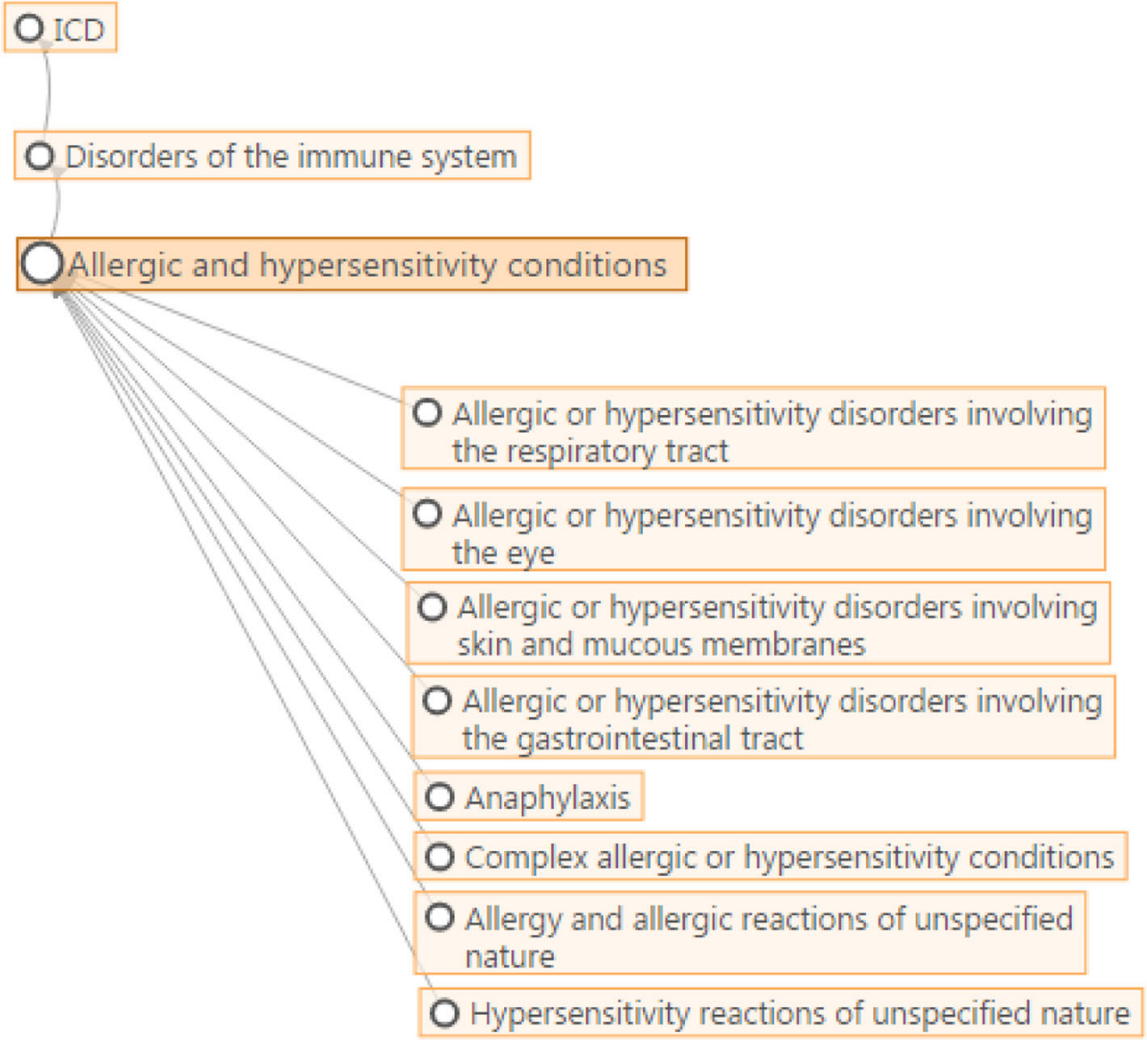
The current “Anaphylaxis” section of the ICD-11 beta draft platform (July 2016 version)

In order to create a more appropriate classification for allergic and hypersensitivity conditions in ICD-11, a detailed action plan was coordinated in 2012 based on scientific evidence for the necessity of change; each step has been documented by peer-reviewed publications [7, 8, 11–19]. The continuing close collaboration between our group and WHO has the backing of the Joint Allergy Academies, composed by representatives of American Academy of Allergy Asthma and Immunology (AAAAI), European Academy of Allergy and Clinical Immunology (EAACI), World Allergy Organization (WAO), American College of Allergy Asthma and Immunology (ACAAI), Asia Pacific Association of Allergy, Asthma and Clinical Immunology (APAAACI), Latin American Society of Allergy, Asthma and Immunology (SLAAI). The main outcome of this process has been the construction of the *Allergic and hypersensitivity conditions* section within the new chapter, *Disorders the Immune System,* which has been incorporated into ICD-11 [16, 20] (Fig. 3). The “Anaphylaxis” sub-section is one of 8 main headings in this new section.

To further inform the allergy community and to ensure that the revision process is transparent as advised in the ICD-11 revision agenda, we report the building process we used for the pioneering “Anaphylaxis” subsection of ICD-11.

## Methods

### Building the “Anaphylaxis” subsection of ICD-11

**Fig. 4:**
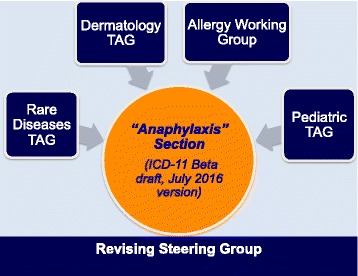
Harmonizing points of views among specialties for the construction of the ICD-11 “Anaphylaxis” section (TAG = Topic Advisory Group)

The design of the anaphylaxis subsection was based on the *Allergic and hypersensitivity diseases* proposal, which had been validated by crowd-sourcing and simplified according to ICD Revision Steering Group (RSG) guidance. The construction of the new *Allergic and hypersensitivity conditions section* of the ICD-11 resulted by intensive scientific, academic and technical discussions to ensure comparability and consistency. The development of the “Anaphylaxis” sub-section involved strong academic input and extensive consultation and agreement from the relevant Topic Advisory Groups (TAGs) and Expert Working Groups (EWGs) (Fig. 4). The intensive exchange of e-mails and teleconference/videoconferences started in February 2014 and was the basis for the submission of proposals into the online ICD-11 beta draft platform. All the actions of the Allergy Working Group have so far been undertaken with RSG guidance.

**Fig. 5:**
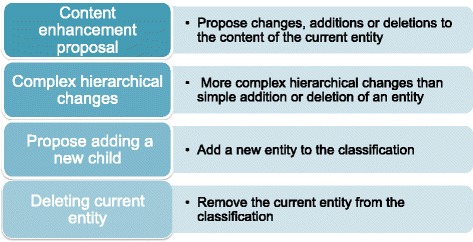
Types of the proposals and its purposes available in the ICD-11 Beta draft

The innovatory electronic authoring and browsing platforms developed for the ICD Revision Project enabled ICD-11 to be assembled and refined in a sophisticated manner including metadata such as definitions, synonyms and causal mechanisms for each concept and support for a complex polyhierarchical structure. One of the most relevant innovations enables proposals for additions or changes to be made online [20]. The system allows for 4 types of proposal: enhancement of content attached to entity (adding or revising metadata), addition of new entity as child of existing entity, deletion of entity and, for more radical proposals, complex hierarchical changes (Fig. 5). Each submitted proposal has to be supported with a rationale and peer-reviewed references, and requires addition of metadata to a standardized template, the “content model”, which includes: title, definition, synonyms, narrower term, exclusions, body system, body site, signs and symptoms, causal agents and causal mechanisms [20].

The ICD-11 beta draft platform [20] can be considered a WHO web-observatory in which the representatives of RSG and TAGs can monitor the proposal submissions and comments. Based on these, the RSG members can approve, partially approve or reject a proposal. Each proposal is then scrutinized by WHO and, if accepted, can then be implemented and incorporated into the current version of ICD-11, following which the change will be visible in the Browser. A proposal may be rejected in part or in full with the reasons for the decision provided by commentaries to the proposal. Alternatively further clarification may be sought from the proposer.

In order to document the construction process fully, we analyzed all of the above actions in order to describe the historic and current status of the *Anaphylaxis* sub-section and analyze the reasons for the principal changes. For this evaluation we considered actions related exclusively to this sub-section and proposals related to the patient’s background (e.g. personal history of anaphylaxis). Some proposals related to the topic were submitted during the revision process in order to adjust the higher ICD-11 hierarchy in general (e.g. removal of all anaphylaxis and allergic or hypersensitivity conditions from the parent *Adverse effects, not elsewhere classified* after the construction of the new section). We did not include proposals not directly related to the patient, such as *Family history of anaphylaxis*.

## Results

### The “Anaphylaxis” sub-section of ICD-11

**Fig. 6:**
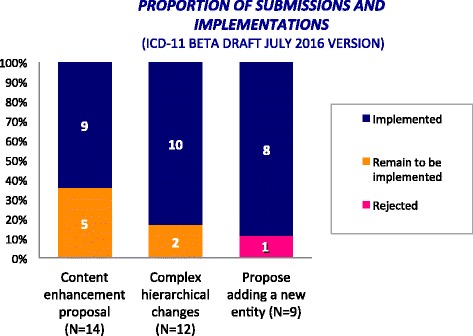
Proposals submitted into the ICD-11 beta draft platform (as at July 2016) for the construction of the new “Anaphylaxis” section

Most of the proposals for the construction of the *Anaphylaxis* sub-section were submitted to the online ICD-11 Beta draft platform in the period from February to March 2015, but the hierarchical structure was adjusted later following additional comments and proposals. During this process, we submitted a total of 35 proposals, of which 25 (71.5%) were content enhancement proposals or complex hierarchical changes. Of the 35 proposals, 27 (77%) were implemented, 7 (20%) remain to be implemented (Fig. 6). The only rejected one was related to the proposal of adding a new entity (*Anaphylaxis classified by clinical severity*). Justifications were provided.

As a result of all the previous actions, the new “Anaphylaxis” sub-section was constructed, with 11 entities classified under 7 main headings: Anaphylaxis due to allergic reaction to food, Drug-induced anaphylaxis, Anaphylaxis due to insect venom, Anaphylaxis provoked by physical factors, Anaphylaxis due to inhaled allergens, Anaphylaxis due to contact with allergens and Anaphylaxis secondary to mast cell disorders.

## Discussion

Allergic and hypersensitivity disorders are managed not only by allergists but also by specialists from a range of different disciplines. As a consequence, intensive scientific and technical discussions with TAGs and EWGs were essential for achieving consensus for the new classification, which will for the first time enable anaphylaxis to be properly represented within ICD.

Since anaphylaxis has never been addressed by a single section in the ICD, it was to be expected that most of the proposals were for content enhancement and complex hierarchical change. These kinds of proposals, in general, support the building processes of new structures. The only partially implemented proposal appeared as such because the incorrect proposal type (deletion of entity) was inadvertently selected for removal of the link to a second parent, which is a hierarchical change. As explained above, the polyhierarchical structure of the ICD-11 Foundation enables any given entity to be linked to more than one parent (Fig. 2).

The only rejected proposal concerned the addition of “Anaphylaxis classified by clinical severity” as a new entity. The reason for the rejection was that ICD-11 enables diagnoses to be linked to a range of parameters by the addition of one or more “extensions” in a process termed post-coordination. WHO has been promoting this classification strategy in which a stem entity (e.g. Anaphylaxis) can be more fully defined by linking it to a range of different value sets including severity, anatomical location and causal agent [20]. All the proposals that have yet to be implemented are related to “personal history of” or to instances where the use of post-coordination would enable the intended meaning to be captured, such as severity grade or allergens and triggers.

Some limitations of the current study may have to be considered. Since the online ICD-11 beta draft is not final and is updated regularly, the current results may not be reproduced if reanalyzed in the future with the same methodology. Although this manuscript presents some technical aspects of classification and new ICD-11 concepts, its aim is also to serve as an introduction to ICD-11 for ICD end-users in the allergy community using anaphylaxis as an example.

The construction of the new section dealing with anaphylaxis means that the latter will now be recognized as a clinical condition requiring specific documentation and management. By allowing all the relevant diagnostic terms for anaphylaxis to be included in the ICD-11 MMS, WHO has recognized their importance not only to clinicians but also to epidemiologists, statisticians, health care planners and other stakeholders. Importantly the new classification will enable the collection of more accurate epidemiological data to support quality management of patients with allergies, better health care planning and decision-making and public health measures to reduce the morbidity and mortality attributable to allergy. Examples are the availability of adrenaline auto-injectors in all countries for patients at risk, the provision of resuscitation kits in public places and the implementation of prevention campaigns in surgical and radiology departments.

The Orphanet, lead by the French National Institution of Health and Medical Research (INSERM) and the French Ministry of Health, is responsible for developing an inventory of rare diseases and a classification system which could serve as a template to update International terminologies. When the WHO launched the revision process of the ICD, a rare diseases TAG was established. So far 5,400 rare diseases listed in the Orphanet database have an endorsed representation in the foundation layer of ICD-11 [21], but anaphylaxis is not yet into the list.

## Conclusion

For the first time, anaphylaxis is now properly classified and has attained greater visibility within ICD. Additionally to all the benefits expected by the actions to update terminology, definitions and classification of allergic and hypersensitivity conditions through the ICD-11 revision, we strongly believe that anaphylaxis is a public health priority and that in order to support awareness and quality clinical management of patients it should therefore be formally added to the list of rare diseases.

## Abbreviations

ICD: International Classification of Diseases
RSG: Revision Steering Group
TAG: Topic Advisory Group
WGs: Working Groups
WHA: World Health Assembly
WHO: World Health Organization
